# Interaction between Thermal Modification Temperature of Spruce Wood and the Cutting and Fracture Parameters

**DOI:** 10.3390/ma14206218

**Published:** 2021-10-19

**Authors:** Luďka Hlásková, Jiří Procházka, Vít Novák, Petr Čermák, Zdeněk Kopecký

**Affiliations:** Department of Wood Science and Technology, Faculty of Forestry and Wood Technology, Mendel University in Brno, 61300 Brno, Czech Republic; jiri.prochazka@mendelu.cz (J.P.); vit.novak@mendelu.cz (V.N.); petr.cermak.und@mendelu.cz (P.Č.); zdenek.kopecky@mendelu.cz (Z.K.)

**Keywords:** thermal modification, machining, cutting force, fracture toughness, shear yield strength, spruce wood

## Abstract

This work examines the effect of thermal modification temperatures in the production of thermally modified wood on the cutting and fracture parameters when cutting heat-treated spruce wood by a circular sawblade machine. The samples were thermally modified at 160, 180, 200, and 220 °C. One sample was unmodified and was used as a reference sample. On the basis of the performed experiments, the fracture parameters (fracture toughness and shear yield strength) were calculated for the axial–perpendicular direction of cutting. In comparison with the theoretical assumptions, the influence of temperature on the cutting and fracture parameters was confirmed. Thermally treated wood is characterized by increased fragility and susceptibility to crack formation, as well as reduced density, bending strength, and shear strength. These properties significantly affect the size of the cutting force and feed force, as well as the fracture parameters. As the temperature increases, the values of these parameters decrease. The mentioned material characteristics could be useful for the optimization of the cutting process, as well as for the issue of energy consumption during the machining of heat-treated wood.

## 1. Introduction

Wood has always been a material that humans use daily, and since it is an available renewable building material, there is a good chance that this will still be true in the future [1]. In civil engineering, wood is most often incorporated in timber structures and timber buildings [2]. Over the last twenty years, it has also been used for facades and terrace floors; however, its organic composition affects these uses rather negatively [3]. This is mainly due to the fact that untreated wood is subject to biological and weathering degradation, to which it is exposed when it is used as an unprotected exterior [4]. However, with advanced industrial methods, such as thermal modification, this situation has largely improved [5]. Since the beginning of the development of thermal modification, heat-treated timber has been a frequent subject of research investigations. Although this is one of many possible wood hydrothermal processing techniques, it is the most frequently used method after kiln drying [6]. By altering the wood structure, which varies depending on the temperature used [7], the thermally treated wood has properties different from those of untreated wood [8]. The most important change is the gradual breakdown of hemicelluloses and, partly, cellulose at the locations of its amorphous structure. This mainly results in a significant reduction in bending strength, with the modulus of elasticity in bending being disproportionately less reduced [9]. An interesting consequence, in terms of mechanical properties, is an increase in hardness [9,10], but this has not always been confirmed [8]. The reduction of density [11] and the esterification of cellulose mean that thermally treated wood has fewer free hydroxyl groups [12], leading to a rapid decrease in the equilibrium moisture content of the wood [13], which is, thus, less susceptible to biodegradation [14]. Changes in the mechanical properties strongly correlate with the increasing temperature used for the modification. Although the weakened thermally modified wood is not suitable for structural purposes [15], it is increasingly used in practice, for example, for facade paneling, terrace floors, and also various elements in the urban space, as its increased biological resistance finds its application there [16]. Unlike physical and mechanical properties, which are described very extensively in the literature, there is a lack of studies focused on the technological properties of heat-treated wood. However, changes in the mechanical and physical properties are reflected in the machining of thermally modified wood [17]. As the density of the thermally modified wood decreases, the cutting force is lowered during machining [18,19], which is also reflected in the reduction in energy consumption [20]. In the past, tests were conducted regarding the effect of thermal modification on the machining force, but they were mostly related to milling [18,21] or grinding [22]. On the other hand, the cutting process has not been given much attention in the area of thermally modified wood. Additionally, new findings have introduced a relatively new method to the machining process, which is based on the cutting-force determination using fracture mechanics. This method was first applied to homogeneous materials, in particular, to metal machining [23]. The method consists of the definition of the fracture toughness and the shear yield strength [24], i.e., unlike conventional methods of calculating the cutting force, this method is not based on the determination of the specific cutting resistance, *k_c_* [25], which is a function of many factors in the case of wood machining. The computational model also uses the application of the Ernst–Merchant theory in the conditions of sawblade timber cutting. The method was later applied to a wide range of materials [26], including wood [27,28,29,30,31], as it can take into account the effect of the altered material structure on the size of the cutting force. Thanks to new knowledge, it is possible to model the machining of different materials more precisely using a prediction that highly correlates with actual machining [32]. This paper calculates the cutting force by the computational model using the fracture methodology, based on the measured data of the feed force and the moment of force during the sawblade cutting of thermally modified spruce wood, under various thermal modifications. Furthermore, the fracture parameters (fracture toughness and shear yield strength) [24], and the methodology for their application to woodworking, were determined [27]. Thus, they loosely follow the previous measurements, in which these parameters were determined in the unmodified wood of beech (*Fagus sylvatica* L.) [28,33], in which beech wood modified in different ways was subjected to experiments. The aim of the research was to examine the effect of the thermal modification temperatures in the production of thermally treated wood on the cutting and feed force during circular saw cutting. Moreover, the fracture parameters (fracture toughness and shear yield strength) were determined directly from the machinability tests.

## 2. Materials and Methods

### 2.1. Materials

**Figure 1:**
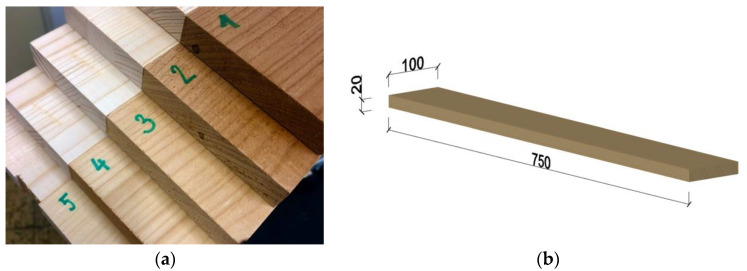
(**a**) (1) temperature 220 °C; (2) temperature 200 °C; (3) temperature 180 °C; (4) temperature 160 °C; (5) a reference sample without TM, (**b**) dimensions of the sample.

Spruce wood (*Picea abies*) samples, taken from the Training Forest Enterprise Masaryk Forest Křtiny (TFE), an organizational part of the Mendel University in Brno (CZ), were used. The samples were oven-dried (at 103 ± 2 °C) before the thermal modification process and were cut from the defect-free boards with standard circular saws. Ten samples for each modification temperature were used for the experiment. The samples were 750 mm long, 20 mm thick, and 100 mm wide (Figure 1b).

Thermal modification at 160 °C, 180 °C, 200 °C, and 220 °C (Figure 1a) was applied in a small-scale laboratory chamber, volume 0.7 m^3^ (KATRES spol. s r.o., Jihlava, Czech Republic), under atmospheric pressure and in a superheated steam environment. The modification phase (at 160 °C, 180 °C, 200 °C, and 220 °C) was maintained for 2 h.

**Figure 2:**
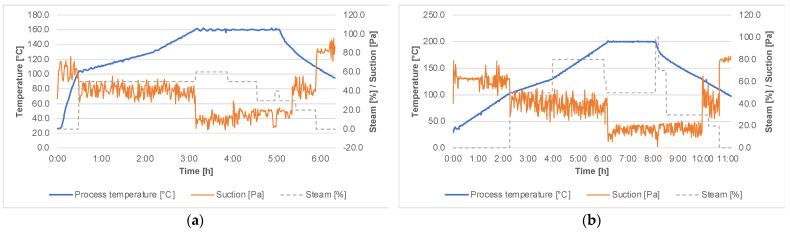
Technological parameters of the modification process of heat-treated wood at 160 °C (**a**), and at 200 °C (**b**).

Figure 2a,b show the records of the thermal modification process (TM) to obtain thermally modified wood at 160 °C and at 200 °C. The intensity and the degree of the modification process were determined by mass loss (ML), based on the oven-dried mass before and after the thermal modification process.

Table 1 presents the results of the weight before and after TM, the density before and after TM, mass loss, and the moisture content. Ten samples for each temperature were used and ten samples were not exposed to the effects of temperature (marked REF). Before and after thermal modification, all samples were conditioned at 20 °C and 65% relative humidity until the equilibrium moisture content was reached. The moisture content was measured by a humidity meter (HMB-WS25, Merlin Technology GmbH, Ried im Innkreis, Austria), which is used for quick nondestructive measurements.

### 2.2. Machinability Test

The machinability tests were performed on a research device focused on cutting with circular sawblades. The test device was placed at the laboratory of the Department of Engineering, the Faculty of Forestry and Wood Technology, Mendel University, in Brno. The test device simulated the conditions of a circular sawing machine in actual operation [34]. During the machinability tests, the moment of force, *M_c_*, and the rotational speed, *n*, were measured using the T34 FN-HBM (Hottinger Baldwin Messtechnik, Darmstadt, Germany) contactless sensor. The sensor was connected to the measuring control unit Spider8 (HBM, Darmstadt, Germany), which feeds and simultaneously processes the output moment of force, *M_c_*, and the rotational speed *n* signals. Spider8 is an electronic measuring system designed for measuring mechanical quantities (force, pressure, travel, speed, relative elongation, etc.) via the connected sensors (active and passive). The control unit communicates with the computer, where the control software assesses the processed signals. The workpiece feed force is measured tensiometrically using the S2-HBM resistive dynamometer (HBM, Darmstadt, Germany), which is located between the ball screw nut and the infeed carriage in such a way that its torsional stress, which would cause measurement inaccuracies, may be avoided. The dynamometer senses tension and pressure up to 100 N or 200 N, with the accuracy class of 0.05.

The circular sawblade for longitudinal wood cutting (Flury Systems AG, Arch, Switzerland), with carbide-tipped straight teeth, was used for the experiment. The parameters of the circular sawblade were as follows: the diameter was *D* = 350 mm; the teeth number was *z* = 28; and the sawblade thickness was *s* = 2.5 mm. The tool geometry was as follows: the clearance angle was *α_f_* = 15°; the rake angle was *γ_f_* = 20°; and the cutting-edge radius was *ρ*_0_ = 8 μm.

The machine settings were as follows: optimum operating rotational speed = 3800 min^−1^ (i.e., the cutting speed *v_c_* = 69.6 m·s^−1^); the feed rate, *v_f_*, varied from 2–22 m·min^−1^ with the steps presented in Table 2. This corresponded to the changing of the mean uncut chip thickness, *h_m_*, and the feed per tooth, *f_z_*. A series of ten measurements were performed for each type of machined material and the present cutting conditions. The values of the cutting forces were subjected to statistical evaluation using a one-factor analysis of variance ANOVA test and a Scheffé test (StatSoft, Hamburg, Germany). Statistical analyses were done for the significance level *α* = 0.05.

For the following calculations, it was necessary to determine the cutting model, which is determined on the basis of the used technology by characterizing the individual angles between the wood fiber grain, the tool planes, and the motion vectors. In the case of the longitudinal cutting of wood with a circular sawblade, this is the axial-perpendicular cutting model. The calculation of the kinematic parameters of circular saw cutting is performed in accordance with the relations presented in Table 2. These variables (*f_z_*, *h_m_*, *φ*_2*m*_) are the input parameters for the calculation, and their values are also shown in Table 2.

**Figure 3:**
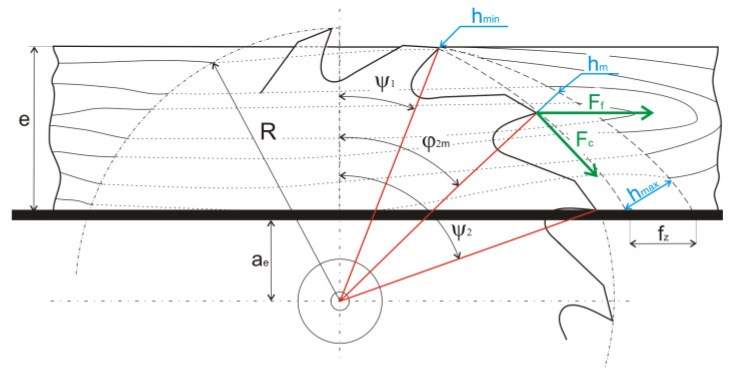
Circular sawing process scheme: *e*—workpiece height; *a_e_*—position of the workpiece; *R*—circular sawblade radius; *ψ*_1_—entry angle; *ψ*_2_—exit angle; *φ*_2*m*_—mean fiber cutting angle; *h_min_*—minimum uncut chip thickness; *h_m_*—mean uncut chip thickness; *h_max_*—maximum uncut chip thickness; *F_c_*—cutting force; *F_f_*—feed force; *f_z_*—feed per tooth.

The circular sawing process scheme (Figure 3) indicates that the angle of fiber cutting varies. At the point of tooth contact with the workpiece, it is equal to the entry angle, *ψ*_1_ (at this point, the uncut chip thickness has the minimum value, *h_min_*). At the point of circular sawblade teeth disengagement, it is equal to the exit angle, *ψ*_2_ (the maximum uncut chip thickness, *h_max_*, is reached). The mean fiber cutting angle, *φ*_2*m*_, is then equal to the average value of both angles. When calculating, the mean uncut chip thickness, *h_m_*, is considered; it is determined at the point of the mean fiber cutting angle, *φ*_2*m*_.

### 2.3. Methodology for the Determination of Fracture Properties from the Machinability Test

The average total cutting power, *P_c_T_*, during cutting with a circular sawblade can be calculated by the means of the cutting forces model presented by [23,24]. This model takes into account the elements of fracture mechanics (fracture toughness *R*) and shear yield strength, *τ_γ_*. This methodology was elaborated for the cases of wood cutting with the frame saw machine by the authors [35,36,37], the band saw machine [29], the milling machine [38], and the circular sawing machine [33].

Because of the material properties included in the model (fracture toughness, *R*, and shear yield strength, *τ_γ_*), this model allows for the determination of the cutting power for wood machining processes, including the inclusion of the wood origin [39], or the type of wood modification [33,40], and its impact on the energy consumption. The model can be presented as Equation (1):

(1) $$P_{c\_T}=P_{c}+P_{ac}+P_{dull}=z_{a}\cdot \frac{\tau_{\gamma }\cdot b\cdot \gamma }{Q_{shear}}\cdot h_{m}\cdot v_{c}+z_{a}\cdot \frac{R\cdot b}{Q_{shear}}\cdot v_{c}+P_{ac}+P_{dull}$$

where *z_a_* is the number of simultaneously cutting teeth; *b* is the kerf width; *γ* is the shear strain along the shear plane; and *Q_shear_* is the coefficient of friction correction.

Total power consists of three components. The first component describes:

1a: The internal work of plasticity along the shear plane, where the shear strain along the shear plane, *γ,* is described as:

(2) $$\gamma =\frac{\cos\gamma_{f}}{\cos\left(\Phi -\gamma_{f}\right)\sin\Phi }$$

where *γ_f_* is the rake angle, and *φ* is the shear plane angle.

Cutting by circular sawblade machine is characterized by a small uncut chip thickness, so it was necessary to adjust the value of the angle of the cutting plane according to the relationship [41] and take into account the dependence of the ratio of the fracture toughness and the shear yield strength.

1b: The internal work required for the separation/formation of new surface, where fracture toughness, *R*, corresponds to the specific work of material separation.

The coefficient of friction correction, *Q_shear_*, can be found in both parts of the first component. *Q_shear_* depends, in principle, on the orientation of the shear plane to the machined workpiece and represents an effect of the friction between the rake face and the separated material. *Q_shear_* is computed according to [23,24]:

(3) $$Q_{shear}=\left[1-\left(\sin\beta_{\mu }\cdot \sin\Phi /\cos\left(\beta -\gamma_{f}\right)\cos\left(\Phi -\gamma_{f}\right)\right)\right]$$

where *β_μ_* = tan^−1^*μ* is the friction angle, and *μ* is the coefficient of friction.

Using the measured moment of force, *M_c_*, and the feed force, *F_f_*, other components of the resulting active force were determined. The calculation was based on the Ernst–Merchant circle force diagram [42].

2: The second component expresses the kinetic energy for chip removal by the circular sawblade (does not affect the value of cutting resistance). The acceleration power of chips, *P_ac_*, can be described as a function of mass flow and tool velocity [26,27,40]:

(4) $$P_{ac}=\dot{m}\cdot v_{c}^{2}$$

(5) $$\dot{m}=\frac{b\cdot l\cdot v_{f}\cdot \rho_{w}}{2}$$

where *l* is the cut length, and *ρ_w_* is the wood density.

All this work is provided externally from the cutting force components moving in parallel to the machined surface [27]. The effect of chip acceleration power, *P_ac_*, on the overall cutting power is negligible. Therefore, *P_ac_* was omitted from the analyses performed in this research.

3: The last component, *P_dull_*, is the power that considers the dulling of cutting edges. It explains the increase in the cutting forces observed throughout the tool life in the real processes [29]. It is important to note that this model assumes perfect cutting-edge sharpness; therefore, the component, *P_dull_*, can be omitted. Moreover, it does not consider the effect of dulling and chip momentum due to the average values of the feed rates during wood cutting [33].

The cutting force is expressed by the line slope in the form:

*F_c_*^1*z*^ = (*k*)∙*h_m_* + *q* (6)

In this case, *k* corresponds to the slope of the linear regression line, and *q* corresponds to the intercept of the linear regression line with the *y* axis. The independent variable of the regression is the mean uncut chip thickness, *h_m_*. It is possible, therefore, to determine the values of the fracture parameters (fracture toughness, *R*_||__⊥_, and shear yield strength, *τ_γ_*_||__⊥_) by comparing the regression (Equation (8)) with the experimental data obtained from the machinability tests. The method of calculating the fracture toughness and shear yield strength is based on the cutting theory originally proposed by [43].

The mathematical procedure for the computation of the shear yield strength and the fracture toughness is expressed in Equations (7) and (8), from the slope of the linear regression line and the intercept, respectively:

(7) $$\tau_{\gamma ∥⊥}=\frac{k\cdot Q_{shear}}{b\cdot \gamma }$$

(8) $$R_{∥⊥}=\frac{q\cdot Q_{shear}}{b}$$

## 3. Results and Discussion

### 3.1. Cutting Force and Feed Force

**Figure 4:**
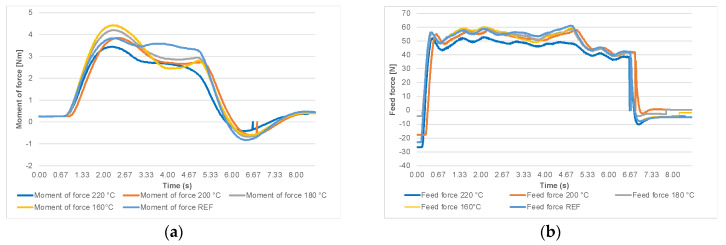
(**a**)The moment of force for feed rate 10 m·min^−1^; (**b**) feed force for feed rate 10 m·min^−1^.

The moment of force and the feed-force curves are shown in Figure 4a,b, where three cutting phases can be distinguished. At the beginning of the cutting process, the moment of force and the feed force rise steeply and reach a maximum value. Then, after a small peak, the signal stabilizes. For the calculation of the mean moment of force and the feed force values, only this so-called steady-state signal range was taken into consideration. This part of the record was further processed, and the results of the experiment and the calculation of the fracture parameters are based on it. The next phase is when the circular sawblade leaves the workpiece. This part of the measurements is accompanied by a steep decrease in forces because the circular sawblade is no longer pushed to overcome the cutting resistance of the workpiece. The forces are stabilized at the values of the so-called passive resistances, caused by friction in the bearings, aerodynamic losses, etc. The values of the passive resistances must be subtracted from the measured values of the moment of force and the feed force.

**Figure 5:**
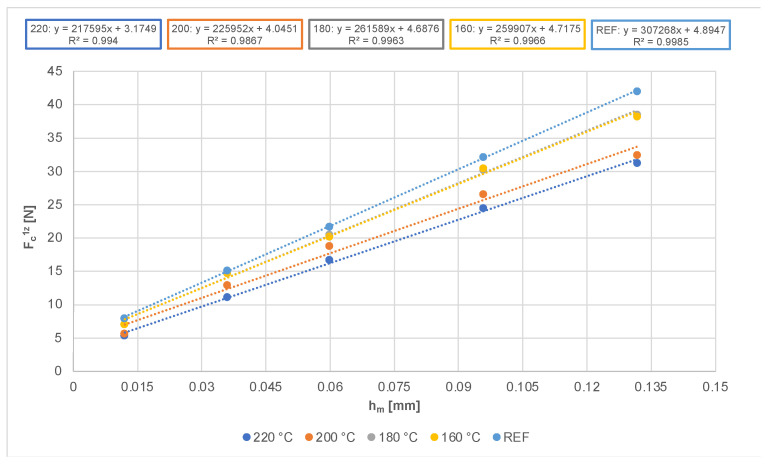
Dependance of average values of cutting force per single tooth on uncut chip thickness.

Figure 5 presents the dependence of the average value of the measured cutting force per single tooth as a function of the mean uncut chip thickness. An almost linear increase in the cutting force occurred, along with an increasing uncut chip thickness. This finding confirms the theoretical assumptions (see Equation (6)). Figure 5 further presents the coefficients of determination, *r*^2^, and the regression equations of the cutting force per single tooth as a function of the uncut chip thickness, with cutting-modified and native spruce, respectively.

The ANOVA analysis was performed for each feed rate separately and the results of the ANOVA test confirm the effect of temperature on the values of the cutting forces (see Table 3). Subsequently, a Scheffé test (a multiple comparison test) was performed at each feed rate. All tests showed statistically significant differences in the mean values of the cutting forces for each feed rate. Therefore, we were able to statistically prove the effect of temperature on the size of the cutting force when cutting spruce wood and thermally modified wood with a circular sawblade.

The graph of the cutting forces per single tooth, dependent on the size of the mean uncut chip thickness (Figure 5), shows that the cutting force increases with an increasing mean uncut chip thickness. This dependence is linear, as confirmed by [44,45,46]. Moreover, the graph indicates the effect of the modification temperature on the value of the cutting force for individual thermally modified wood samples and for the unmodified spruce sample. Figure 5 clarifies that modified wood behaves differently from unmodified spruce samples during machining.

The results show that the cutting force is the highest when the unmodified sample (REF) is machined. We also found that the higher the temperature when modifying wood, the lower the value of the cutting force. This corresponds with the results of the authors of [47], who confirmed a decrease in the cutting force during the milling of pine samples (*Pinus contorta*). They observed the highest decrease in cutting force for a modification temperature of 240 °C (26.9%). The authors of [18] also confirmed that, at temperatures above 160 °C, a lower cutting force is needed for milling thermally modified pine wood (*Pinus sylvestris*) than for the milling of unmodified pine wood. Finally, [48] confirmed that the cutting force for milling spruce decreases with higher temperatures of modification. The decrease in the cutting force is caused by the lower strength of the thermally modified wood [49,50,51,52,53,54,55].

The authors of [56] conducted research into heat-treated birch wood and demonstrated a decrease in the modulus of rupture (MOR) with an increasing modification temperature (especially above 200 °C). The authors of [57] concluded that the MOR of spruce wood modified at 220 °C was, on average, 50% lower than that of unmodified wood. Wood treated with thermal modification becomes more fragile, and its bending strength and tensile strength decrease by up to 10–30%, based on the research of [58]. The decrease is related to the loss of chemicals in the wood [59,60,61,62]. This leads to a decrease in the wood weight and wood density [53,54,60,63,64]. Our samples showed a 3.17% decrease in the density of wood modified at 220 °C, compared to untreated wood. The mass loss is caused by the thermal degradation of the wood cell wall components (especially hemicelluloses) [63,64]. Hemicellulose plays an important role in reducing the physical and mechanical properties of thermally modified wood because it is less stable to heat than cellulose and lignin at higher temperatures [65,66]. The authors of [67] state that the physical properties of hemicellulose change under modification temperatures between 127 °C and 235 °C, the properties of lignin change from 167 °C to 217 °C, and those of cellulose change from 231 °C to 253 °C. It follows that the higher the modification temperature, the more the individual wood structure components will be degraded, accompanied by a decrease in the mechanical and technological properties of the wood. Thermal modification also causes an increased Young’s module, fragility, and a susceptibility to crack formation [67,68,69], as well as a decrease in the specific cutting resistance and the specific work of fracture [18,69,70,71]. The cutting force, at machining, of wood modified at 220 °C and a feed rate of 22 m·min^−1^ is 25% lower than that of the unmodified sample, and in wood treated at 200 °C, it is 21.6% lower. The decrease in the cutting force for wood modified at 160 °C and 180 °C was minimal, and the cutting force was 8% lower compared to the unmodified sample. The results of the research of [72] show that the milling of heat-treated wood is less energy-intensive, and also reveal that the cutting force decreased only slightly when the modification temperature increased from 160 °C to 180 °C. With a further increase in temperature, the cutting force decreased significantly, as in our measurement. Thanks to the altered physical and mechanical properties of heat-treated wood (e.g., higher fragility), chips are easier to break and crumble, which has a positive effect on reducing the cutting force.

**Figure 6:**
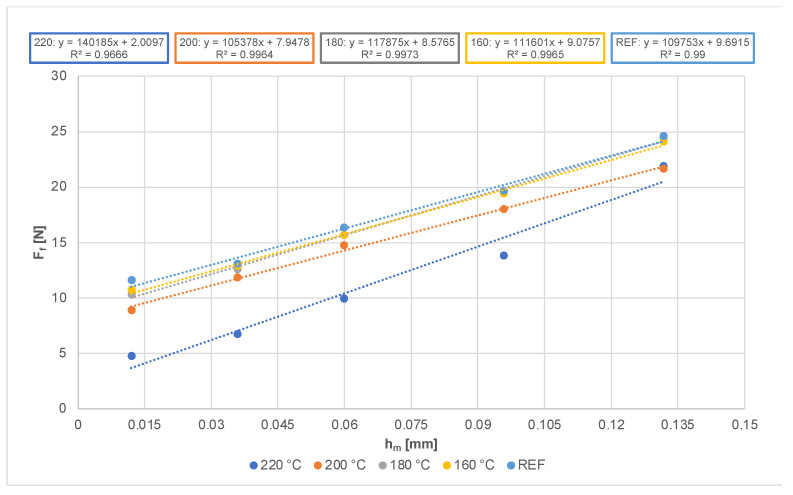
Dependance of average values of feed force on uncut chip thickness.

Figure 6 shows that the force required to feed the workpiece in the sawblade at a speed of 2–22 m·min^−1^ ranges from 5 N to 25 N for the experiments performed. In general, the feed force increases for all modified materials, as well as for the reference sample, with an increasing feed rate. The dependence is linear with a relatively high coefficient of determination. In the research of [73], a positive correlation was observed between the feed rate and the feed force during the cutting of particle-board panels using a circular saw.

The graph also shows that, as the modification temperature increases, the feed force decreases. The most significant difference is obvious in the machining of wood thermally modified at 220 °C and the lowest feed rate (2 m·min^−1^); the decrease compared to the reference sample was almost 60%. The difference between the reference sample and the material modified at 160 °C and 180 °C was up to 10%. Due to the easier splitting and fragility of thermally modified wood, and thanks to its deteriorated mechanical properties, the feed mechanisms do not need such a large feed force, as is the case with untreated wood.

### 3.2. Fracture Parameters: Fracture Toughness and Shear Yield Strength

In Figure 5, linear regression lines are drawn and the coefficients of determination, *r^2^*, for each model, and the regression equations, are presented. On the basis of the experiments performed (from the regression parameters), the characteristic input parameters (*φ*, *μ*, *β_μ_, γ*, *Q_shear_*) entering the model for the axial-perpendicular model were calculated for cutting with the circular sawblade for the kerf width, *b* = 3.5 mm, and for a tooth position defined by the mean fiber cutting angle, *φ*_2*m*_ = 36.5°. The characteristic data for all machined materials were estimated according to [24]. The input values are shown and compared in Table 4.

**Figure 7:**
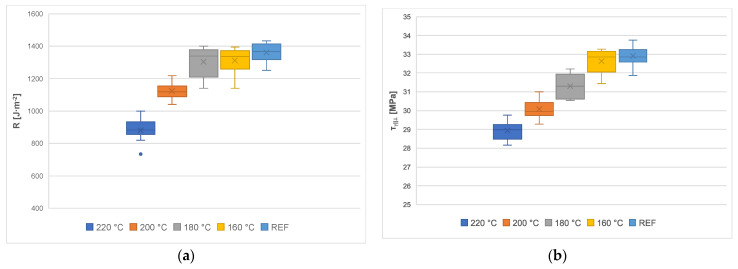
(**a**) Fracture toughness, (**b**) shear yield strength.

The determination of the main parameters of the model was performed on the basis of regression analysis. Fracture toughness, *R*_||__⊥_, was determined from the intercept, and the shear yield strength, *τ_γ_*_||__⊥_, from its slope. The average values of fracture toughness, *R*_||__⊥_, and shear yield strength, *τ_γ_*_||__⊥_, were calculated with their corresponding standard deviations (Table 5). The values of the shear yield strengths, *τ_γ_*_||__⊥_, were calculated for uncut chip thickness, *h_m_* > 0.13 mm, when the cutting resistance was practically constant [74]. The values of these fracture parameters are presented and compared in Table 5 and Figure 7a,b.

Figure 7a,b summarize the fracture toughness and shear yield strength in boxplots to illustrate the influence of the modification temperature on these fracture parameters. The comparison of the results between the fracture properties, given in Table 5 and Figure 7a,b, shows that the fracture toughness and shear yield strength of the thermally modified wood is not only dependent on density, but also on the modification temperature and its influence on the changes in the internal structure and the degradation of cell wall components.

Fracture toughness and shear yield strength are listed in the literature for individual load directions and for the main directions of cutting or crack propagation. However, our measured results represent a combination of these basic directions because the machining was performed in the axial-perpendicular direction of cutting. The values of the fracture parameters are relevant only for a given cutting edge direction, which is characterized by the mean fiber cutting angle, *φ*_2*m*_, and therefore cannot be considered as the material constants.

With regard to wood modified at higher temperatures, a decrease in shear yield strength was observed, compared to unmodified wood (the decrease in shear yield strength at 160 °C is approximately 1%, at 180 °C it is 4.9%, at 200 °C it is 8.6%, and at 220 °C it is 12.1%, compared to REF). The same trend can be observed for the fracture toughness. As the modification temperature increases, the fracture toughness decreases (at 160 °C by 3.6%, at 180 °C by 4.2%, and at 200 °C by 17.4%). The wood modified at a temperature of 220 °C even shows up to 35% lower values of fracture toughness than unmodified wood. Thus, again, the higher the modification temperature, the higher the degradation of the mechanical properties of the wood.

These results are in general agreement with the professional literature and confirm that thermal modification negatively affects the mechanical properties of wood. This claim can be explained by the loss of material within the cell lumen, and the degradation of hemicellulose by the effect of high temperatures during modification [64,74,75]. Some anatomical changes in the structure of modified wood may also contribute to the reduction in the mechanical properties. For example, [76] noticed cracks between the tracheids in heat-treated softwood species. The authors of [77] found cracks in the middle lamella and layer S1 in spruce wood modified at temperatures from 180 °C to 200 °C. Furthermore, deformations of the libriform fibers and collapsed vessels in the wood were observed. These changes in the wood structure can lead to significantly different load behavior.

So far, only a few studies have been published on the fracture properties of modified wood. For example, [78] presents the fracture properties of unmodified spruce and spruce treated by thermal modification and acetylation; the authors note a 20% reduction in the fracture toughness of acetylated wood, and a 50–80% reduction in the heat-treated spruce wood. The authors of [33] published the fracture properties of modified beech wood. Their results show that the fracture properties of the modified materials depend not only on density, but also on the type of modification, cell wall degradation, and the internal structure. In the work of [79], the authors focus on the various drying techniques and their influence on the fracture parameters. On the basis of their research, they conclude that an increase in the wood drying temperature and, thus, its thermal modification, lead to higher fragmentation and graininess of the resulting sawdust during machining. This indicates a large change in the fracture properties of the wood thus treated. The results of [80] show reduced values of fracture toughness and fracture energy. The experiments of [81], conducted on heat-treated spruce, show that the fracture energy decreases linearly with an increasing mass loss, suggesting that the thermal degradation of wood affects the fracture energy. The microstructure and nanostructure within the cellular structure can, therefore, be changed by thermal modification [81]. The reduction in the fracture toughness of thermally modified wood can also be attributed to its lower plastic ductility. According to [82], the fracture energy is higher in the case of unmodified wood compared to thermally modified wood. Crack initiation is easier, and the crack propagation phase consumes less energy and takes place in heat-treated wood in a more fragile way. The authors of [83] observed the effects of thermal modification on the fragility of wood (*Styrax tonkinensis*) and state that the main factor influencing fragility was the loss of hemicellulose and the amorphous region of cellulose due to degradation. The authors of [13] also claim that the wood fragility increases with the deterioration of the fracture properties caused by the loss of amorphous polysaccharides. The results of these studies show that the fracture properties of thermally modified wood will be negatively affected, and this is supported by our results.

Because of the fibrous structure, wood has different shear yield strengths in three perpendicular directions. Determining the conditions of clean shear in wood is very difficult. From the perspective of machining, our method of violating the workpiece is most closely approached by shear in the transverse plane, where the forces act perpendicularly to the fibers in the tangential or radial direction. This method of violation is often called "cutting fibers" or "shear strength" [53]. Table 5 and Figure 7b present the comparison of the shear yield strengths. The trend is similar to that of fracture toughness, although not to such an extent. An increasing temperature of modification leads to a reduced shear yield strength. Our results are in line with the study by [75], where a decrease in shear strength was found after thermal modification. The explanation lies in the (partial) transformation of polyoses into furfural polymers. Such degradation of the hemicelluloses probably has a negative effect on shear yield strength. On the other hand, lignin is the main component of the middle lamella, which plays an important role in shear strength [67]. Increased networking within the lignin polymer network could have a positive effect on shear yield strength. Therefore, the deterioration of shear yield strength observed is not as large as that of other mechanical properties.

## 4. Conclusions

The basic relationships for calculating the fracture characteristics of heat-treated spruce samples (fracture toughness and shear yield strength) were derived from the cutting tests without the need to perform complex fracture tests. The fracture parameter values are suitable only for the axial-perpendicular cutting model and, therefore, cannot be considered material constants.

On the basis of the measurements, it can be concluded that thermal modification affects the mechanical, physical, and technological properties of wood. Thermally treated wood is characterized by increased hardness, fragility, and susceptibility to crack formation, as well as reduced density, bending strength, and shear strength. These properties significantly affect the size of the cutting force and feed force, as well as both parameters of the calculation model: fracture toughness, *R*_||__⊥_, and shear yield strength, *τ*_γ||__⊥_. As the temperature increases, the values of these parameters decrease.

Knowledge of these parameters is essential for the correct estimation of the size of cutting forces using a computational model based on fracture mechanics, since optimizing the cutting process, and the issue of energy consumption during the machining of heat-treated wood, are very important when considering the overall economy of production and the production process in the woodworking and other associated industries. This computational model differs from conventional methods for calculating the cutting force, especially by its possible application to a wide range of materials, including modified materials, which have a modified internal structure, or wood-based composite materials.

Comparing the results of the fracture parameters with the literature is very difficult, since no similar work has been carried out to determine the fracture parameters based on cutting tests. In addition, the work of fracture calculated in this way consists, not only of the fracture energy (as determined in the splitting tests), but also the friction and compression properties of the cut material. The fracture energy achieved by the splitting tests is, therefore, lower than the work of fracture obtained by the cutting tests.

## Figures and Tables

**Table 1:** Characteristics of samples.

Sample	Weight before TM [g]	Weight after TM [g]	Density before TM [kg·m^−3^]	Density after TM [kg·m^−3^]	Mass Loss [%]	Moisture Content [%]
REF	286.47 ± 35.12	Same as before TM	416.03 ± 50.95	Same as before TM		7.64
160 °C	285.26 ± 31.55	283.91 ± 30.71	413.85 ± 45.77	411.90 ± 44.55	0.47 ± 0.256	7.12
180 °C	288.77 ± 33.04	285.54 ± 32.44	418.94 ± 47.94	414.26 ± 47.06	1.12 ± 0.185	6.89
200 °C	286.78 ± 32.26	279.76 ± 31.34	416.06 ± 46.80	405.88 ± 45.47	2.45 ± 0.425	7.25
220 °C	283.04 ± 31.88	274.08 ± 28.90	411.63 ± 46.25	397.63 ± 41.93	3.17 ± 0.847	7.16

**Table 2:** Kinematic parameters and machine settings.

*v_f_* [m·min^−1^]	$v_{f}=f_{z}\cdot n\cdot z$	2	6	10	16	22
*f_z_* [mm]	$f_{z}=\frac{v_{f}}{n\;.\;\;z}$	0.019	0.056	0.094	0.15	0.207
*h_m_* [mm]	$h_{m}=f_{z}\;.\;\sin\varphi_{2m}$	0.011	0.033	0.055	0.089	0.123
*v_c_* [m·s^−1^]	$v_{c}=\pi \cdot D\cdot n$	69.6				
*φ*_2*m*_ [°]	$\varphi_{2m}=\frac{\psi_{1}+\psi_{2}}{2}$	36.5				
*ψ*_1_ [°]	$\psi_{1}=arccos\left(\frac{a_{e}+e}{D/2}\right)$	31.0				
*ψ*_2_ [°]	$\;\psi_{2}=arccos\left(\frac{a_{e}}{D/2}\right)$	42.0				

**Table 3:** ANOVA analysis: influence of temperature on the cutting force per single tooth.

*v_f_* [m·min^−1^]	*F*	*p* Value	*F_krit_*	Evaluation of Statistical Tests	
2	146.74	0.00002	5.19	*F* > *F_krit_*	Statistically significant differences
6	19.25	0.00307	5.19	*F* > *F_krit_*	Statistically significant differences
10	38.78	0.00059	5.19	*F* > *F_krit_*	Statistically significant differences
16	16.48	0.00438	5.19	*F* > *F_krit_*	Statistically significant differences
22	24.09	0.00183	5.19	*F* > *F_krit_*	Statistically significant differences

**Table 4:** Comparison of regression equation, coefficient of determination values, slope values, and intercept values.

	Regression Equation	Coefficient of Determination *r*^2^	Slope Value *k* [N·m^−1^]	Intercept Value *q* [N]
REF	*F_c_*^1*z*^ = 307,268 *h_m_* + 4.8947	0.9985	307.268	4.8947
160 °C	*F_c_*^1*z*^ = 259,907 *h_m_* + 4.7175	0.9966	259.907	4.7175
180 °C	*F_c_*^1*z*^ = 261,589 *h_m_* + 4.6876	0.9963	261.589	4.6876
200 °C	*F_c_*^1*z*^ = 225,952 *h_m_* + 4.0451	0.9867	225.952	4.0451
220 °C	*F_c_*^1*z*^ = 217,595 *h_m_* + 3.1749	0.994	217.595	3.1749

**Table 5:** Comparison of fracture parameters.

	μ [-]	β_μ_ [°]	φ [°]	γ [-]	Q_shear_ [-]	*τ_γ_*_||__⊥_ [MPa] Mean Score ± SD	R [J·m^−2^] Mean Score ± SD
REF	0.264	14.798	37.600	2.068	0.797	32.9 ± 0.543	1359.64 ± 57,208
160 °C	0.154	8.744	40.628	2.024	0.868	32.62 ± 0.644	1310.42 ± 79,349
180 °C	0.158	8.962	40.519	2.024	0.865	31.29 ± 0.613	1302.11 ± 90,881
200 °C	0.086	4.906	42.547	2.007	0.921	30.08 ± 0.536	1123.64 ± 56,797
220 °C	0.402	21.904	44.048	1.480	0.716	28.93 ± 0.487	881.92 ± 71,852

## Data Availability

https://doi.org/10.6084/m9.figshare.16828507.v1 (accessed on 19 October 2021).

## Nomenclature

*α*	significance level
*α_f_*	clearance angle
*β_μ_*	friction angle
*γ*	shear strain along the shear plane
*γ_f_*	rake angle
*μ*	the coefficient of friction
*ρ*_0_	cutting-edge radius
*ρ_ω_*	wood density
*τ_γ_*	shear yield strength
*τ_γ_*||_⊥_	shear yield strength for axial-perpendicular model
*φ*	shear plane angle
*φ*_2*μ*_	mean fiber cutting angle
*ψ*_1_	entry angle
*ψ*_2_	exit angle
*a_e_*	position of the workpiece
*b*	kerf width
*D*	diameter
*e*	workpiece height
*F_c_*	cutting force
*F_c_* ^1*z*^	cutting force per single tooth on uncut chip thickness
*F_f_*	feed force
*f_z_*	feed per tooth
*h_m_*	mean uncut chip thickness
*h_max_*	maximum uncut chip thickness
*h_min_*	minimum uncut chip thickness
*k*	slope of the linear regression line
*k_c_*	specific cutting resistance
*l*	cut length
*M_c_*	moment of force
*ML*	mass loss
*MOR*	modulus of rupture
*n*	rotational speed
*P_ac_*	acceleration power of chips
*P_c_T_*	average total cutting power
*P_dull_*	power which considers the dulling of cutting edges
*q*	intercept of the linear regression line
*Q_shear_*	coefficient of friction correction
*R*	fracture toughness
*R*_||__⊥_	fracture toughness for axial-perpendicular model
*r* ^2^	coefficient of determination
*REF*	reference sample
*s*	sawblade thickness
*SD*	standard deviation
*TM*	thermal modification process
*v_c_*	cutting speed
*v_f_*	feed rate
*z*	teeth number
*z_a_*	number of simultaneously cutting teeth
